# Anterior retrosplenial cortex is required for long-term object recognition memory

**DOI:** 10.1038/s41598-020-60937-z

**Published:** 2020-03-09

**Authors:** Ana Belén de Landeta, Magdalena Pereyra, Jorge H. Medina, Cynthia Katche

**Affiliations:** 1 Universidad de Buenos Aires, Facultad de Medicina, Buenos Aires, Argentina; 2 CONICET-Universidad de Buenos Aires, Instituto de Biología Celular y Neurociencia “Dr. Eduardo De Robertis” (IBCN), Buenos Aires, Argentina

**Keywords:** Cortex, Long-term memory

## Abstract

The retrosplenial cortex (RSC) is implicated on navigation and contextual memory. Lesions studies showed that the RSC shares functional similarities with the hippocampus (HP). Here we evaluated the role of the anterior RSC (aRSC) in the “what” and “where” components of recognition memory and contrasted it with that of the dorsal HP (dHP). Our behavioral and molecular findings show functional differences between the aRSC and the dHP in recognition memory. The inactivation of the aRSC, but not the dHP, impairs the consolidation and expression of the “what” memory component. In addition, object recognition task is accompanied by c-Fos levels increase in the aRSC. Interestingly, we found that the aRSC is recruited to process the “what” memory component only if it is active during acquisition. In contrast, both the aRSC and dHP are required for encoding the “where” component, which correlates with c-Fos levels increase. Our findings introduce a novel role of the aRSC in recognition memory, processing not only the “where”, but also the “what” memory component.

## Introduction

During the last few years, the interest on the retrosplenial cortex (RSC) role in cognition has increased. Several human and non-human animal studies have demonstrated its involvement in spatial navigation^1–4^, physical^5–7^ and temporal^8^ contextual memory, the “when” component of a short-term recognition memory^9^, associations between multiple sensory stimuli^10^, prospective thinking as part of the default mode network^11,12^ and memory extinction^13,14^. The RSC comprises the totality of rodents posterior cingulated cortex and its most caudoventral subdivision in primates^15^, and it is highly connected with brain structures involved in memory processing, such as the hippocampus (HP)^16,17^. In humans as well as in rodent models, both HP and RSC lesions have shown similar memory impairments^18^. In this context, an interaction has been shown between the HP and the RSC during a persistent memory formation using an aversive context-dependent task^19^. Moreover, HP inactivation leads to decreased function of the RSC^20,21^.

Despite their high interconnectivity, the RSC and the HP deploy diverse connections to different structures suggesting that they may operate in distinct ways. The RSC major connections to the visual cortex and the claustrum^22,23^ suggest that it could be involved in memories with a huge dependence on the visual pathway, such as the “what” component of recognition memory. Particularly, recognition memory allows distinguishing between familiar or novel places, objects and individuals; therefore it is necessary for an individual’s performance throughout life. This memory is an episodic memory subtype that is formed by three components: “what”, “where” and “when”^24^. The integration of these components creates a unique memory. In addition, the RSC is connected with the perirhinal cortex (PRh)^16^, the prime structure involved in object recognition memory^25–27^. Despite the potential role of the RSC in recognition memory, its precise involvement in the “what” memory component encoding remains unknown.

In the present study we evaluated the role of anterior RSC (aRSC) in the processing of the different components of recognition memory and contrasted its function with that of the dorsal HP (dHP). We used three variants of the object recognition task, with different amount of spatial information to evaluate the “what” component with or without “where” component associated as well as the explicit “where” component of the recognition memory. All these variants have only one sample phase (training session), which allows us to distinguish the different memory stages (acquisition, consolidation and retrieval) by using a transient brain region inactivation method. Here we demonstrated that aRSC is required for the formation and the expression of two of the main features of recognition memory the “what” and the “where” components. Also, we differentiated the mnemonic functions of the aRSC from those of the dHP, given that the last one is only required for the “where” component of recognition memory.

## Results

### aRSC, but not dHP, is required for the “what” memory component processing

**Figure 1:**
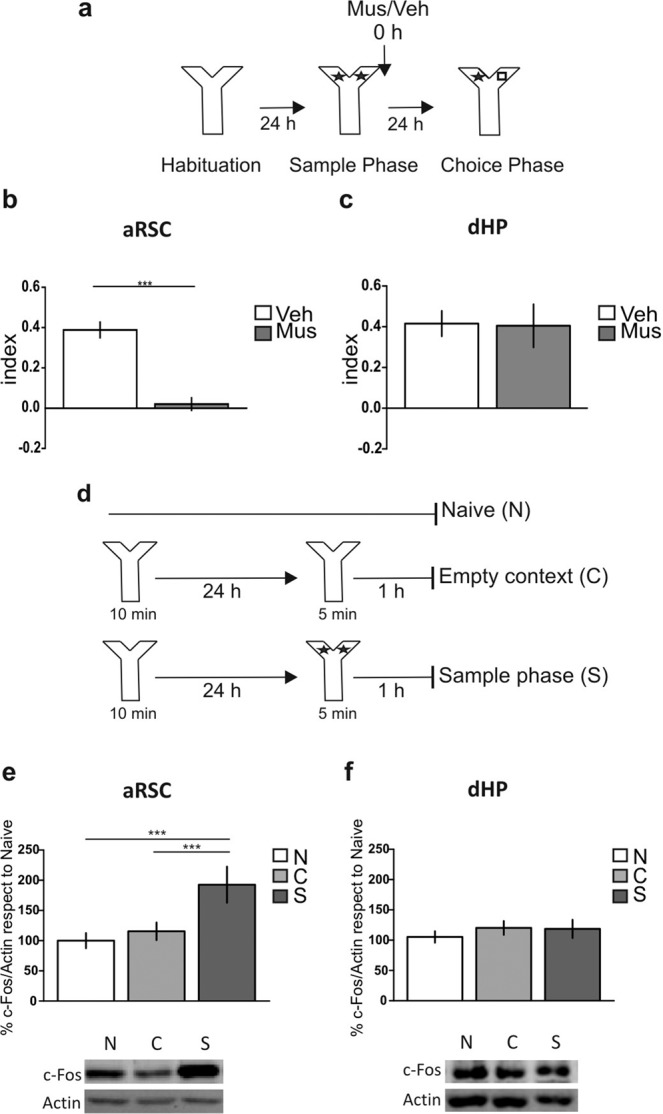
Functional dissociation between the anterior retrosplenial cortex and the dorsal hippocampus in the “what” memory component processing. (**a**) Schematic representation of infusions and the Y-maze object recognition (Y-OR) behavioral paradigm. (**b,c**) Animals were infused with Muscimol (Mus, gray bar) or Vehicle (Veh, white bar) into the anterior retrosplenial cortex (aRSC) (**b**) or dorsal hippocampus (dHP) (**c**) immediately after sample phase; the choice phase was performed 24 h later. Data are expressed as memory index mean ± SEM. **p < 0.01 Mus vs. Veh; Two-tailed Student’s t-test. (**b**) n = 8 per group, (**c**) n = 4–5. (**d**) Schematic representation of the behavioral protocol. (**e,f**) Percentage of c-Fos in the aRSC (**e**) and the dHP (**f**) of naïve animals (N, white bar), and animals subjected to the empty context (**c**, light gray bar) or to the sample phase (S, gray bar) 1 h after the Y-OR. Representative blots are shown below each graphic. Data are expressed as mean ± SEM of c-Fos percentage respect to naïve. ***p < 0.005, Bonferroni Multiple Comparison Test after two-way ANOVA. (**e**) n = 4, (**f**) n = 3–5.

To make a comparative study about the participation of aRSC and dHP in the “what” component of the recognition memory, we decided to study object recognition memory in the non-spatial Y-maze (Y-OR) task^28^. Due to the high walls, small corridors and the lack of explicit clues in the Y-maze, the Y-OR task has minimal spatial information and therefore a negligible “where” component. First, we validated the Y-OR task by transient inactivation of the PRh, confirming its requirement for object recognition memory (Supplementary Fig. S1; p = 0.0031, t = 3.774, df = 11; Muscimol vs. Vehicle, Student’s t test, n = 5–7). Then, we analyzed the participation of the aRSC and dHP in the formation of Y-OR long-term memory (LTM) by infusing the GABA_A_ receptor agonist muscimol (0.1 μg/μl) or vehicle (saline) into the aRSC or the dHP immediately after the sample phase (training session), and we evaluated memory expression 24 h later during the choice phase (Fig. 1a). We found memory impairment associated to the aRSC inactivation (Fig. 1b; p < 0.0001, t = 6.611, df = 14; Muscimol vs. Vehicle, Student’s t test, n = 8 per group). In contrast, muscimol infusion into the dHP did not affect the Y-OR memory expression (Fig. 1c; p = 0.9278, t = 0.094, df = 7; Muscimol vs. Vehicle, Student’s t test, n = 4–5). To further evaluate the participation of these structures in Y-OR memory formation, we studied the levels of c-Fos expression, an immediately early gene that is a transcription factor usually upregulated in active brain structures^29^, following a sample phase. Consistent with our behavioral data, c-Fos levels increased 1 h after the Y-OR sample phase in the aRSC (Fig. 1d; Bonferroni after two-way ANOVA_(2,17)_, F_(interaction)_ = 0.31, p = 0.7347, F_(group)_ = 9.86, p = 0.0014, n = 4*per group*) but not in the dHP (Fig. 1e; two-way ANOVA_(2,26)_, F_(interaction)_ = 0.27, p = 0.8977, F_(group)_ = 1.68, p = 0.2056, n = 3–5). Full-length blots are shown in the Supplementary Fig. S2.

**Figure 2:**
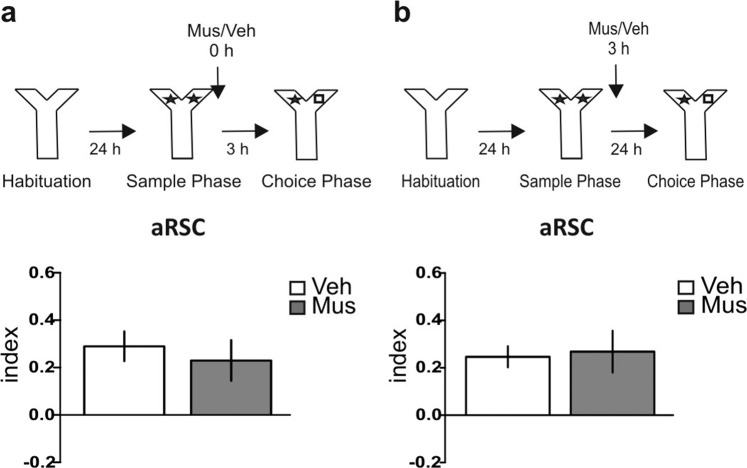
aRSC inactivation does not affect short-term memory and its requirement in consolidation is time-dependent. (**a**) *Upper panel:* Schematic representation of infusions and behavioral paradigm. *Bottom panel:* Animals were infused with Mus (gray bar) or Veh (white bar) into the aRSC immediately after the sample phase. Choice phase was performed 3 h later to assess short-term memory. Data are expressed as memory index mean ± SEM. Musvs. Veh; Two-tailed Student’s t-test. n = 5–8. (**b**) *Upper panel:* Schematic representation of infusions and behavioral paradigm. *Bottom panel:* Animals were infused into the aRSC with Mus (gray bar) or Veh (white bar) 3 h after sample phase; choice phase was performed 24 h after sample phase. Data are expressed as memory index mean ± SEM. Mus vs. Veh; Two-tailed Student’s t-test. n = 6 per group.

Next, we studied whether the inactivation of the aRSC induced long-term Y-OR amnesia by impairing the consolidation process, responsible of the stabilization of the lasting memory, or by directly affecting memory processing. Thus, we infused muscimol in this structure immediately after the sample phase, but this time we evaluated the short-term memory (STM) expression by performing the Y-OR test session 3 h later. We found that muscimol infusion did not affect STM expression (Fig. 2a; p = 0.6298, t = 0.496, df = 11; Muscimol vs. Vehicle, Student’s t test, n = 5–8). In addition, we studied the time window during which the aRSC was required for the Y-OR LTM consolidation and found that muscimol infusion 3 h after training did not affect memory expression 24 h later (Fig. 2b; p = 0.8304, t = 0.220, df = 10; Muscimol vs. Vehicle, Student’s t test, n = 6 per group). Thus, aRSC is specifically required during the first moments after the sample phase to allow the formation of a lasting Y-OR memory without affecting the formation of the STM.

**Figure 3:**
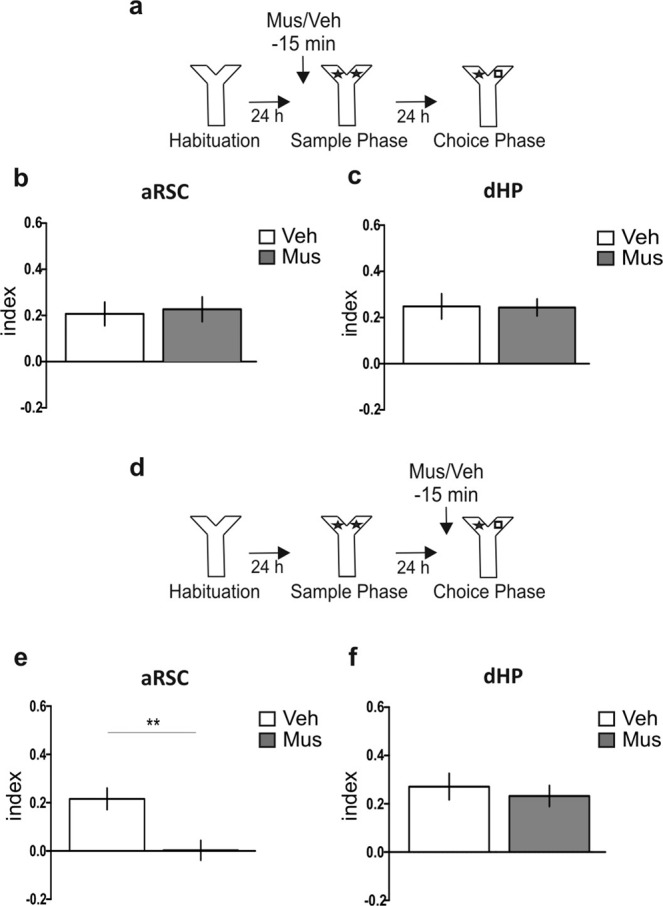
aRSC is required for object recognition memory expression, but not for its acquisition. (**a**) Schematic representation of infusions and behavioral paradigm. (**b,c**) Animals were infused with Mus (gray bar) or Veh (white bar) into aRSC (**b**) or dHP (**c**) 15 min before sample phase; choice phase was performed 24 h later. Data are expressed as memory index mean ± SEM. Mus vs. Veh; Two-tailed Student’s t-test. (**b**) n = 6–8, (**c**) n = 7–8. (**d**) Schematic representation of infusions and behavioral paradigm. (**e,f**) Animals were infused with Mus (gray bar) or Veh (white bar) into the aRSC (**e**) or the dHP (**f**) 15 min before choice phase, performed 24 h after the sample phase. Data are expressed as memory index mean ± SEM. *p < 0.05 Mus vs. Veh; Two-tailed Student’s t-test (**e**) n = 10–12, (**f**) n=8–9.

Then, we studied and compared the requirement of the aRSC and dHP in Y-OR acquisition. To achieve this, we inactivated either structure 15 min before the sample phase and tested for Y-OR memory 24 h later (Fig. 3a). Unexpectedly, the inactivation of the aRSC during acquisition had no effect on LTM expression (Fig. 3b; p = 0.9201, t = 0.102, df = 12; Muscimol vs. Vehicle, Student’s t test, n = 6–8). The same result was observed when the dHP was inactivated (Fig. 3c; p = 0.9409, t = 0.076, df = 13; Muscimol vs. Vehicle, Student’s t test, n = 7–8). Thus, neither the aRSC nor the dHP are required for memory acquisition.

In addition, we also investigated the role of these structures during Y-OR memory retrieval by locally infusing muscimol 15 min before choice phase, performed 24 h after sample phase (Fig. 3d). Here, we found that the inactivation of aRSC resulted in an impairment of memory expression (Fig. 3e; p = 0.0022, t = 3.532, df = 19; Muscimol vs. Vehicle, Student’s t test, n = 10–11). On the contrary, when we inactivated the dHP during the choice phase, recognition memory remained intact (Fig. 3f; p = 0.5775, t = 0.569, df = 15; Muscimol vs. Vehicle, Student’s t test, n = 8–9). Therefore, aRSC but not dHP is necessary for retrieving object recognition memory.

### aRSC participates of the “what” memory trace only if is active during memory acquisition

**Figure 4:**
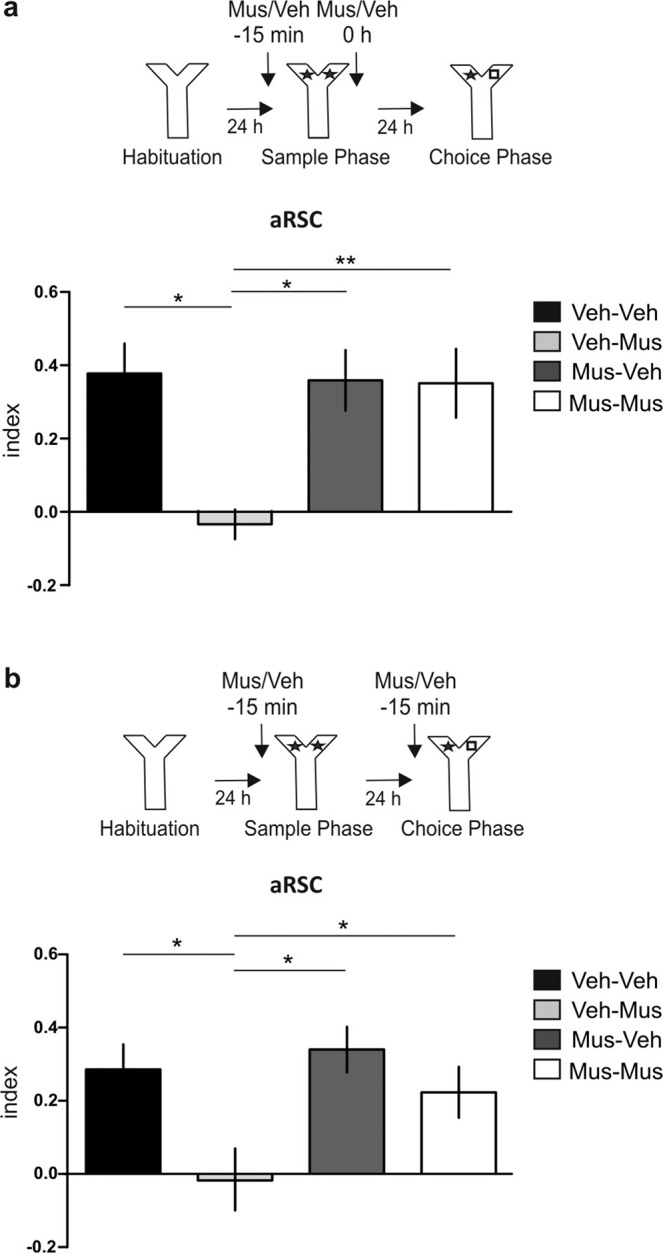
aRSC is recruited to process object recognition memory, only if it is active during memory acquisition. (**a**) *Upper panel:* Schematic representation of infusions and behavioral paradigm. *Bottom panel:* Control group was infused with Veh (black bar) 15 min before and immediately after the sample phase. Rats were infused with Mus 15 min before the sample phase and with Veh immediately after the sample phase (light gray bar) to inactivate the aRSC during memory acquisition. Rats were infused with Veh 15 min before the sample phase and with Mus immediately after the sample phase (gray bar) to inactivate the aRSC during memory consolidation. The last group was infused with Mus both before and after the sample phase (white bar). Data are expressed as mean ± SEM. *p < 0.05, **p < 0.01, Newman Keuls Multiple Comparison Test after one-way ANOVA. n = 5–9. (**b**) *Upper panel:* Schematic representation of infusions and behavioral paradigm. *Bottom panel:* Control animals were infused with Veh (black bar) 15 min before sample and choice phases. Animals were infused with Mus 15 min before the sample phase and with Veh 15 min before the choice phase (light gray bar) to inactivate the aRSC during memory acquisition. Animals were infused with Veh 15 min before the sample phase and with Mus 15 min before the choice phase (gray bar) to inactivate the aRSC during memory retrieval. The last group was subjected to a double-inactivation of the aRSC, animals were infused with Mus before sample and choice phase (white bar). Data are expressed as mean ± SEM. *p < 0.05, Newman Keuls Multiple Comparison Test after one-way ANOVA. n = 9–12.

Since muscimol infusion into the aRSC 15 min before the sample phase did not affect LTM, while its infusion immediately after sample phase did affect it, we hypothesized that the inactivation of the aRSC before sample trial prevents its involvement in the processing of the task. Therefore, we decided to evaluate whether inactivating the aRSC during acquisition interfered with its recruitment in memory processing. To do so, we subjected animals to a double-inactivation of the aRSC, in order to affect acquisition and consolidation (Fig. 4a) or acquisition and retrieval (Fig. 4b). Consistent with the findings described before (Figs. 1b and 3b), we observed that muscimol impaired LTM expression when it was infused into the aRSC after, but not before, the sample phase. However, when the animals were infused with muscimol before and after the sample phase, the latter infusion was unable to impair Y-OR memory expression at the next day (Fig. 4a; Newman-Keuls after one-way ANOVA_(3,28)_, F_(group)_ = 3.788, p = 0.0213, n = 5–9). Analogous findings were observed by interfering with the acquisition and retrieval processes. While the infusion of muscimol before the choice phase impaired memory expression (confirming results of Fig. 3e), this impairment was not observed if the animals were also infused with muscimol before the sample phase (Fig. 4b; Newman-Keuls after one-way ANOVA_(3,38)_, F_(group)_ = 4.386, p = 0.0096, n = 9–12). Our results demonstrate that, aRSC is required for both, consolidation and expression, as far as the structure remains active during acquisition, thus suggesting that muscimol infusion before the sample phase would disengage the aRSC from memory processing.

### aRSC and dHP participation does not depend on contextual cues for object recognition memory formation

**Figure 5:**
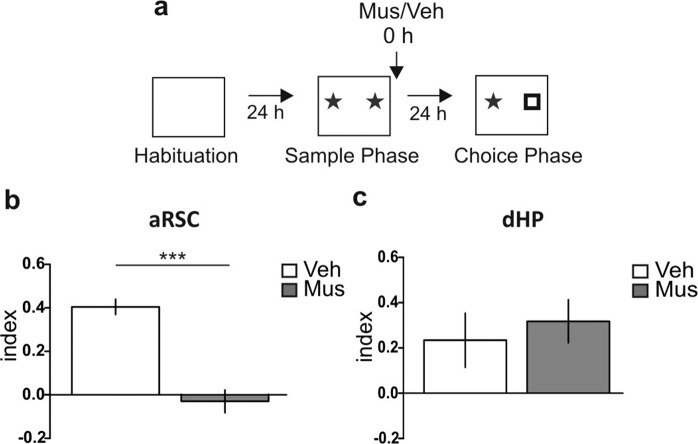
aRSC, but not dHP, is required in spontaneous object recognition task. (**a**) Schematic representation of infusions and spontaneous object recognition (SOR) behavioral paradigm. (**b,c**) Animals were infused with Mus (gray bar) or Veh (white bar) into the aRSC (**b**) or the dHP (**c**) immediately after sample phase, memory was tested 24 h after. Bars represent the memory index mean ± SEM. **p < 0.01 Mus vs. Veh; Two-tailed Student’s t-test. (**b**) n = 7–6, (**c**) n = 7–9.

Then, we decided to evaluate whether the differential role of aRSC and dHP in the recognition memory shown above was due to the high predominance of the “what” component in the Y-OR task. Thus, we replaced the Y-maze for a larger arena with several explicit cues on its walls, and therefore high content of spatial information. Then we evaluated the participation of these structures in the consolidation of the object memory in this spontaneous object recognition (SOR) task by locally infusing either muscimol or vehicle after the sample phase, we tested the memory 24 h later (Fig. 5a). Once again, despite the presence of explicit cues, we found a differential role of the two structures being SOR-LTM impaired when muscimol was infused into the aRSC (Fig. 5b; p < 0.0001, t = 7.059, df = 11; Muscimol vs. Vehicle, Student’s t test, n = 7–6), but not when it was delivered into the dHP (Fig. 5c; p = 0.9378, t = 0.079, df = 14; Muscimol vs. Vehicle, Student’s t test, n = 7–9). Since the role of dHP in processing SOR task is controversial, and taking into account the hypothesis that the dHP participates of the object recognition memory when the animal has acquired enough information about the object^30^, we decided to corroborate our results by increasing the exploration time during sample phase. There were no differences in the memory indexes between rats infused with muscimol or vehicle into the dHP immediately after an 8 min sample phase (Supplementary Fig. S3; p = 0.7886, t = 0.274, df = 12; Muscimol vs. Vehicle, Student’s t test, n = 7 per group).

### Both the aRSC and the dHP encode the “where” memory component

**Figure 6:**
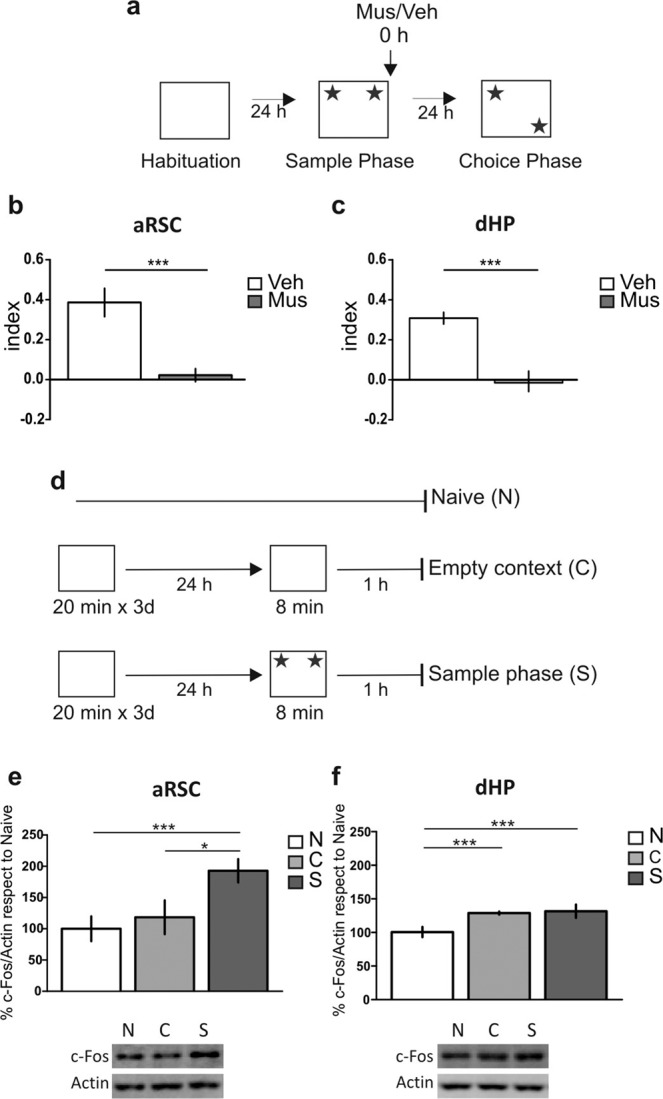
RSC and HP are required in object location performance. (**a**) Schematic representation of infusions and object location (OL) behavioral paradigm. (**b,c**) Rats were infused with Mus (gray bar) or Veh (white bar) into the aRSC (**b**) or the dHP (**c**) immediately after sample phase; memory was evaluated at 24 h. Data are expressed as memory index mean ± SEM. *p < 0.05 Mus vs. Veh; Two-tailed Student’s t-test. (**b**) n = 8 per group, (**c**) n = 11–12. (**d**) Schematic representation of the behavioral protocol. (**e,f** ) Percentage of c-Fos in the aRSC (**e**) and the dHP (**f**) of naïve animals (N, white bar), and animals subjected to the empty context (C, light gray bar) or to the sample phase (S, gray bar) 1 hour after the OL. Representative blots are shown below each graphic. Data are expressed as mean ± SEM of c-Fos percentage respect to naïve. *p < 0.05, ***p < 0.005, Bonferroni Multiple Comparison Test after two-way ANOVA. (**e**) n = 4–5. (**f**) n = 4–5.

Finally, we decided to analyze the role of aRSC and dHP in the processing of the “where” component of recognition memory. To do so, we repeated the previous experiment in the same context, but this time, instead of changing one of the objects during the choice phase, both objects remain the same but one of them was moved to a novel location. Thus, in this object location (OL) task, the “what” component remained constant, but the “where” (location) component was explicitly changed (Fig. 6a). In this case, we found that muscimol infusion immediately after the sample phase into the aRSC (Fig. 6b; p = 0.0003, t = 4.703, df = 14; Muscimol vs. Vehicle, Student’s t test, n = 8 per group) or the dHP (Fig. 6c; p < 0.0001, t = 5.557, df = 21; Muscimol vs. Vehicle, Student’s t test, n = 11–12) induced a memory impairment. In line with these results c-Fos levels were increased in the aRSC (Fig. 6d; Bonferroni after Two-way ANOVA_(2,20)_, F_(interaction)_ = 0.85, p = 0.4419, F_(group)_ = 6.42, p = 0.0070, n = 4–5) and the dHP (Fig. 6e; Bonferroni after Two-way ANOVA_(2,35)_, F_(interaction)_ = 0.87, p = 0.5288, F_(group)_ = 7.54, p = 0.0019, n = 4–5) 1 h after OL sample phase. Full-length blots are shown in Supplementary Fig. S4.Thus, both structures participate in the consolidation of the “where” component of this recognition memory.

## Discussion

In this study we showed that aRSC and dHP are differentially involved in the processing of the “what” and the “where” components of the recognition memory. Our results in the non-spatial Y-OR task demonstrate that the transient aRSC inactivation during memory consolidation and retrieval, but not during memory acquisition, results in LTM expression impairment. Moreover, the inactivation of aRSC after the contextual SOR training also impairs LTM. Data of both tasks confirm the role of aRSC in the “what” memory component processing. We also observed that aRSC is required for memory consolidation and retrieval only when it is completely functional during memory acquisition. As expected, we observed that aRSC is required for memory consolidation in the OL task. In addition, we contrasted aRSC and dHP function for both “what” and “where” memory components. Our results show a differential role of these structures in recognition memory; while the aRSC is involved in encoding both “what” and “where” components, the dHP is only required for the “where” component of memory encoding.

Most of the studies have focused on RSC function in navigation, in part because of the connections between the RSC and the visual cortex and the presence of head-direction, spatial view and place cells in the RSC^31–33^. Some human studies using positron emission tomography and functional magnetic resonance imaging showed an activation of the RSC during episodic memory encoding and retrieval^34–36^. We demonstrated a novel role for the aRSC in the “what” memory component processing, and also corroborate the aRSC requirement in the “where” memory component encoding.

Although some studies indicate that the RSC is not required in object recognition memory, it should be noticed that they were performed using lesions of the whole structure and testing memory in delays no longer than 30 min after the sample phase, i.e. STM^37–40^. Using the Y-OR task enabled us to study the “what” component of the recognition memory, in a context of negligible “where” component (due to the lack of contextual cues), by explicitly changing one object during the test session. Thus, in combination with transient aRSC inactivation, we observed that the functionality of this structure was specifically required after acquisition, during the first moments of the consolidation phase, to grant the stabilization of a lasting memory, but not for the formation of STM that was evident even 3 h after the sample phase. In this way, the role of the aRSC in object recognition memory is similar to that observed for the PRh by inactivating or using lesions on it^26,41–44^. On the contrary, despite being usually associated to the HP due to their high connectivity as well as similar functional outputs^18^, our results show that in contrast to the aRSC, the HP is not involved in Y-OR memory consolidation.

Some studies support the idea that the HP is not necessary for formation and retrieval of the object recognition memory^28,41,45–49^. Others, performed in tasks with strong object-context or object-object association suggest the contrary^26,39,50–54^. These controversial findings about hippocampal-dependent object recognition memory may be explained by differences in behavioral task procedures, species used, and reversible vs. irreversible inactivation methods may explain those controversial findings^30^. Here, we added complexity through the use of a SOR task, in which the “what” component of recognition memory was explicitly studied by changing the object during the choice phase, but with a strong presence of information about the context and location of the object in the environment (“where” component). Our findings showed that the aRSC is still required during the consolidation phase in order to form a lasting memory about the object in the context of a strong “where” component. However, despite the presence of this “where” component, the inhibition of the HP function did not impair memory consolidation. Indeed, letting the dHP to acquire more information about the object/context relation^30^ by increasing the exploration time during the sample phase, also did not recruit this structure for an object recognition LTM.

However, when we evaluated the recognition memory in the OL task we observed that both aRSC and dHP are required for the consolidation to occur. In the OL task the same objects are used during sample and choice phases keeping the “what” component constant. However, the “where” component is explicitly evaluated by moving one of those objects into a new location during the choice phase^37^. Thus, our results show that the aRSC and dHP are required to consolidate the “where” component of recognition memory^18,55,56^. These results are consistent with previous findings showing that both RSC and HP are structures highly specialized in processing spatial information but at the same time put into evidence the differential role played by these structures in processing distinct components of the recognition memory.

In addition to the behavioral analysis, we studied the involvement of aRSC and dHP in recognition memory by measuring the levels of c-Fos, which is used as a marker of neuronal activation^57–59^. In line with our behavioral results, showing that aRSC is required for the consolidation of the “what” and “where” components of recognition memory and the dHP only for the “where” component, we observed that c-Fos levels increased in the aRSC, but not in the dHP, after Y-OR sample phase and in both structures after the OL training. This findings are consistent with the work of Mendez and collaborators, which shows an increase in c-Fos levels in the PRh but not in the HP after a SOR task, and in the HP after OL^55^. Also, they match to a wide range of experiments indicating that c-Fos is associated with the formation of different memories processed in different brain regions^19,29,60–63^. Thus, the increase in c-Fos levels during the consolidation phase of Y-OR memory provides further evidence of the involvement of aRSC in the processing of the “what” component of recognition memory.

Another major finding of this work is that transient inactivation of the aRSC before the sample phase does not affect memory acquisition, but memory consolidation and retrieval are disrupted by the aRSC inactivation immediately after the sample phase or before choice phase, respectively. Here we demonstrate that this occurs because when aRSC is active during memory acquisition, then it is also required for memory consolidation and retrieval; however if aRSC is inactive during the acquisition phase, then it is no longer required for memory consolidation and its retrieval. This effect may be due to a functional compensatory plasticity from others structures implicated in object recognition memory^64–67^. Thus, when the aRSC is not active during acquisition, other brain structures may take control of memory processing. It would be of interest to further analyze how this functional compensation is induced. These results also suggest the importance of using transient inactivation studies in the analysis of brain structures function. Despite the huge advances reached through experiments involving brain lesions, it should be taken into consideration that they impede to distinguish between memory stages and also the behavioral results observed might be due to compensatory effect from other brain structures^64,68^.

Recently, the RSC participation in the “when” memory processing has been reported, since its lesion produces impairment in a short-term object recency memory^9^. This data and our results indicate that the RSC is required to process the different components of recognition memory. In this regard it is important to mention that RSC is one of the structures implicated in Alzheimer disease (AD)^69,70^. The impairment of navigational memory in AD patients^71^ is attributable to the neurodegeneration of different brain regions^72^, including theRSC^73–75^. Another symptom of the AD is the loss of recognition memory^76,77^. Given that our results show the requirement of the aRSC in recognition memory retrieval, possibly part of the loss of recognition memory in AD patients could be explained by the RSC neurodegeneration.

**Figure 7:**
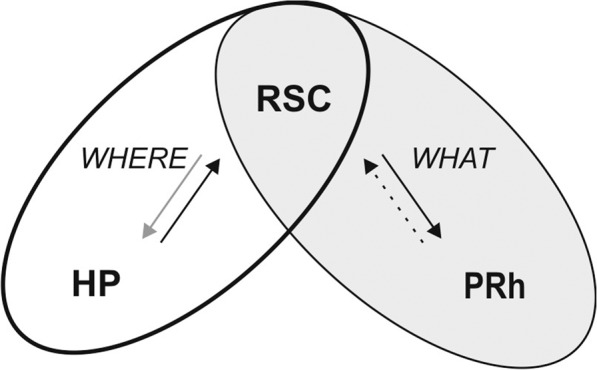
Proposed circuits for the “what” and “where” encoding. The aRSC participates of both “what” and “where” components of recognition memory. Different circuits are recruited to form each memory component; the “what” circuit might be formed by the PRh and aRSC, among other structures. On the other hand, the “where” circuit might be formed at least by the dHP and aRSC. Arrows represent the connectivity between structures; Black arrows represent direct pathways, dotted line symbolizes a possible connection from PRh to aRSC. Gray arrow represents an indirect pathway.

Recognition memory is of fundamental importance for our everyday life. It is supported by networks of interdependent brain regions involving the medial temporal lobe and prefrontal cortex^26,48,78^ which are selectively activated depending on the type and features being recognized. Recognition memory of single items needs the integrity of the PRh while recognition of an object and its spatial location requires also the HP and medial prefrontal cortex functions^27,79–82^. We found that the aRSC functional integrity is required during recognition LTM consolidation in all variants of the task we used. Surprisingly, object recognition memory formation still takes place even when the aRSC is inactive during acquisition, resulting in a RSC-independent LTM. In conclusion, the RSC has a dual role in recognition memory, which might be supported by two circuits formed by the RSC and structures of the medial temporal lobe (Fig. 7). The circuit encoding the “what” component could be formed by the RSC and PRh, which might receive part of the object information from the RSC projections^16^. In accordance with our results, there is evidence that the RSC, HP and postrhinal cortex form the “where” circuit^43,83^. Our results shed light on the knowledge of the RSC function and its dual role in the “what” and “where” processing, and attain to differentiate this structure function from that of the HP.

## Materials and Methods

### Subjects

2.5-month-old male Wistar rats (Facultad de Ciencias Exactas y Naturales, UBA, Argentina) weighting 220–280 g. Animals were housed in groups of five per cage and maintained under 12 h light/dark cycle (lights on at 7:00 am) at 21–23 °C with water and food *ad libitum*. Experimental procedures followed the guidelines of the USA National Institutes of Health Guide for the Care and Use of Laboratory Animals and were approved by the Animal Care and Use Committees of the University Buenos Aires (CICUAL).

### Surgery

Rats were implanted under deep ketamine/xylazine anesthesia (40 and 2 mg/kg, respectively) with 22 G guide cannula in the aRSC at AP −3.9, L ±0.5, DV −1.8 (Supplementary Fig. S5A), the dHP at AP −3.9, L ±3.0, DV −3.0 (Supplementary Fig. S5B) and the PRh at AP −5.5, L ±6.6, DV −7.0 (Supplementary Fig. S5C), coordinates in mm from Bregma according to the atlas of Paxinos and Watson^84^. The cannulas were fixed to the skull with dental acrylic. Obturators were then inserted into the cannula to prevent blockage. After four or five days of recovery from surgery, the animals were gently handled once a day for 2 days and then trained in the recognition task.

### Drug infusion

We infused the GABA_A_ receptor agonist muscimol (Sigma Aldrich, USA) at a dose of 0.1 μg per side into the aRSC, the dHP or the PRh15 min before or immediately after the sample phase or 15 min before choice phase to study memory acquisition, formation or retrieval, respectively^85^. Drug was dissolved in sterile saline. In all cases, infusions were bilateral and had a volume of 1 µl, except PRh infusion which volume was of 0.5 μl. The entire infusion procedure took around 4 min, the infusion rate was 1 µl/min. Injectors were left in place for an additional minute following infusion before they were removed carefully to avoid backflow.

### Cannula placement

Cannula placement was verified after the end of the behavioral procedures by infusions of 1 µl (into the aRSC and the dHP) or 0.5 µl (into the PRh) of a solution of 4% methylene blue in saline. Histological examination of cannula placements was performed. Only the behavioral data from animals with the cannula located in the intended site were included in the final analysis (285 of 330 animals; 14% of the animals were discarded).

### General behavioral task

Recognition paradigm is based on rodents natural behavior to explore novel objects or contexts^86^. In order to study which components of recognition memory the aRSC is processing we used three variants of recognition memory paradigm (Supplementary Fig. S6).

In all tasks there was a habituation session, in which the animal was able to explore the context. One day after the habituation there was a sample phase, consisting in letting each animal explore two identical objects, made of glass, metal or plastic. During the choice phase, 3 or24 h after sample phase, the animal was allowed to explore two different objects or locations, one from the sample phase (familiar object or location) and the other novel, the novel object or location positions were selected by chance and were counterbalanced between animals. During both sample and choice phases we used manual timers to score the time the rodent spent exploring (sniffing or touching) the objects. The experimenters that scored the exploration time were blinded to the animals’ treatment and novel object/location. We calculated the novel object discrimination index as exploration time of the novel object or the novel location of the object minus the exploration time of the familiar object or the familiar location of the object divided the total exploration time. Indexes greater than zero were considered as indicators of memory whereas no preference was considered when the indexes were not significantly different to zero. We analyzed data from animals that had a minimal exploration time of 15 s per object during the sample phase and showed no preference for any side, and during the choice phase explored more than 10 s at least one of the objects (total exploration time for each experiment is showed in Supplementary Table T1). The objects and apparatus were cleaned with a solution of soap, alcohol and water before being presented to each animal.

### Y-shape object recognition

Object recognition was conducted in a Y-shaped acrylic maze^28^. Each arm of the maze was 27 cm length and 10 cm wide, with white walls 40 cm high, preventing the animal to visualize any external cue. The arms in which the objects were placed were shortened to 8.5 cm by guillotine doors. In the habituation session rats were allowed to explore the empty maze during 10 min for one day. During sample phase rats were placed in the start arm and led explored for 5 min two identical objects, placed in each arm of the apparatus. Choice phase consisted in letting the rat explore two different objects for 3 min, one familiar and the other novel (Supplementary Fig. S6A).

### Spontaneous Object Recognition

The task was adapted from Ennaceur and Delacour^86^. The apparatus consisted in an acrylic maze of 40 × 60 ×50 cm, with a transparent acrylic frontal wall while the back and lateral walls were of white acrylic with different cues on it. In the habituation session rats were allowed to explore the empty maze during 15 min for 2 days. Exposition time during sample and choice phases were the same as the Y-OR, with the difference that in this task the objects were located facing each other in the middle of the maze (Supplementary Fig. S6B).

### Object Location

OL task was performed in the same apparatus that the SOR. This task was adapted from Ennaceur and collaborators^37^. The habituation session consisted in letting the rat explore the empty apparatus during 20 min for 3 days. During the sample phase two identical objects were presented to the rat, each one located close to opposite walls of the maze. Rats were allowed to explore the objects and context for 8 min. Choice phase lasted 3 min during which the rat could freely explore the objects and the maze; both objects were familiar, but one of them was located in a novel place (Supplementary Fig. S6C).

### Immunoblot Assays

Animals were divided into three groups: naïve, habituated to the maze or submitted to the Y-OR or OL task. Naïve (N) animals were handled, but they were never exposed to the contexts or objects. A group of animals were exposed to the empty context (C), for 10 min during 1 day and the next day during 5 min for Y-OR, or 20 min during 3 days and 8 min the next day for OL. Sample phased animals (S) were habituated to the empty context for 1 day during 10 min and the next day were exposed to two identical objects for 5 min for Y-OR or were habituated to the empty context 3 days during 20 min and the next day were exposed to two identical objects for 8 min for OL. For both tasks rats were sacrificed by decapitation 1 h after the exposition to the empty context or objects. An experimenter dissected both aRSC and dHP over a plate on ice. The dissection took less than 4 min and tissue was stored at −80 °C until its homogenization.

Tissue from the aRSC and the dHP was homogenized in ice-chilled buffer (pH 7.4, 20 mM Tris-HCl, 0.32 M sucrose, 1 mM EDTA, 1 mM EGTA, 1 mM PMSF, 10 μg/ml aprotinin, 15 μg/ml leupeptin, 10 μg/ml bacitracin, 10 μg/ml pepstatin, 15 μg/ml trypsin inhibitor, 50 mMNaF, 1 mM sodium orthovanadate and 0.1 mM ammonium molybdate). The homogenates were centrifuged at 900 *g* for 10 min to obtain nuclear fractions, the pellets were resuspended in ice-chilled buffer (pH 7.4, 20 mMTris-HCl, 20 mMNaF, 0.2 mM EDTA, 0.5 mM sodium orthovanadate)^87^. Samples were stored at −80 °C until used. Samples of nuclear fractions (7.5 μg of proteins, determinate by Pierce BCA Protein Assay Kit, Thermo Scientific USA) were subjected to SDS-PAGE (polyacrylamide 10%)^62^. Proteins were transferred to Polyvinylidene (PVDF) membranes overnight at 4 °C. Membranes were incubated first with anti-c-Fos antibody^61,62^ (1:1000, Rabbit monoclonal (4), Santa Cruz Biotechnology, USA), then stripped and incubated with anti-Actin antibody (1:25000, Goat monoclonal (C-11), Santa Cruz Biotechnology, USA). Antigen-antibody complexes were visualized by a fluorescent method using ECF substrate (GE Healthcare Life Sciences, USA) and fluorescence-measuring equipment (STORM scanner, GE Healthcare Life Sciences, USA). Densitometry analysis was performed using Gel-Pro Analyzer (version 4.0 Media Cybernetics, USA), optical density (OD) values were obtained for each sample for both c-Fos and Actin. Each c-Fos OD value was normalized to its corresponding Actin OD value, and then normalized to N condition.

### Data analysis

Behavioral data were analyzed by unpaired Student’s t test or one-way analysis of variance (ANOVA) followed by Newman-Keuls post-hoc test. Immunoblots were analyzed by two-way ANOVA followed by Bonferroni post-hoc test. We used Graph Pad Prism 5 (Graphpad, USA) and Infostat (Córdoba National University, ARG). In all cases α level was set at 0.05. All data are presented as mean ± SEM.

## Supplementary information

Supplementary Information.

## Data Availability

The data that support the findings of this study are available from the corresponding author upon reasonable request.
