# Supplementary material for: Anterior retrosplenial cortex is required for long-term object recognition memory
Source: Sci Rep. 2020 Mar 9;10:4002. doi: 10.1038/s41598-020-60937-z (PMC7062718; doi:10.1038/s41598-020-60937-z)
Supplement: Supplementary file 1 — Supplementary Information. [file 41598_2020_60937_MOESM1_ESM.pdf]

**Anterior retrosplenial cortex is required for long-term object recognition memory**

Ana Belén de Landeta[1,2], Magdalena Pereyra[1,2], Jorge H. Medina[1,2] and Cynthia Katche[1,2,*]

[1]Universidad de Buenos Aires. Facultad de Medicina. Buenos Aires, Argentina.

[2] CONICET-Universidad de Buenos Aires. Instituto de Biología Celular y Neurociencia "Dr. Eduardo De Robertis" (IBCN). Buenos Aires, Argentina.

*Corresponding author: ckatche@fmed.uba.ar

Full address, telephone number:

Laboratorio de Memoria, IBCN, Facultad de Medicina, Universidad de Buenos. Paraguay 2155, 3er piso, (C1121ABG), Ciudad Autónoma de Buenos Aires, Argentina. Phone: +54 115285-2800 (ext 53179 or 53180).

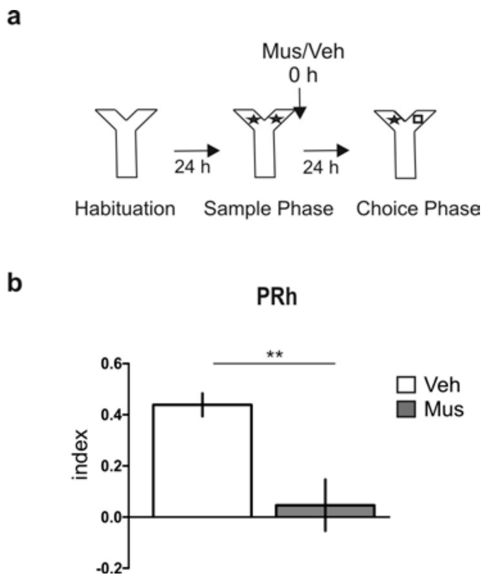


**Figure S1: Transient inactivation of the perirhinal cortex impaired object memory.** (A) Schematic representation of infusions and behavioral paradigm. (B) Animals were infused with Mus (gray bar) or Veh (white bar) into the PRh immediately after the non-spatial Y maze object recognition sample phase and performed choice phase 24 h later. Data are expressed as memory index mean ± SEM. **p< 0.01Musvs. Veh; Two-tailed Student's t-test. n= 6-7.

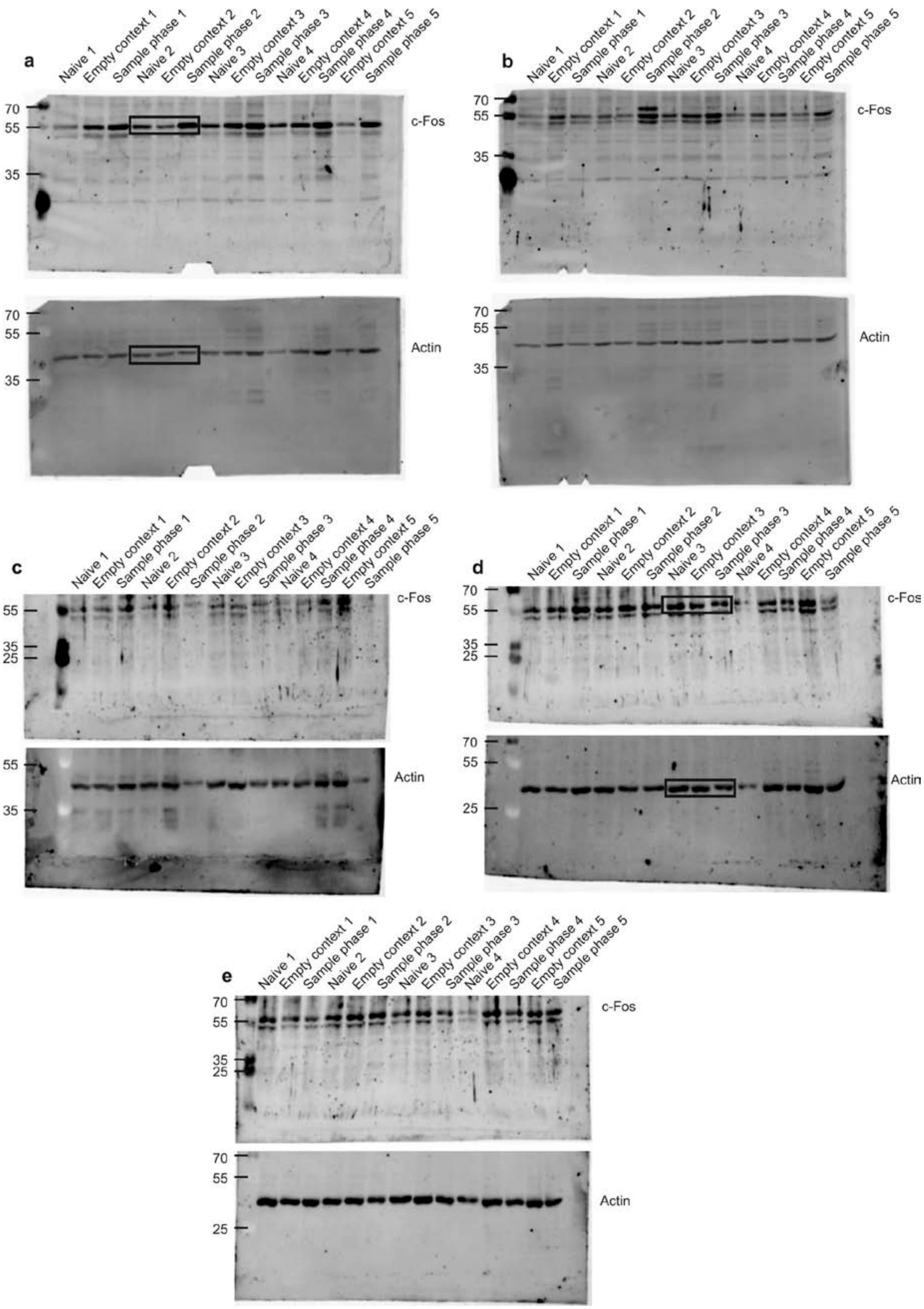
a
Naive 1
Empty context 1
Sample phase 1
Naive 2
Empty context 2
Sample phase 2
Naive 3
Empty context 3
Sample phase 3
Naive 4
Empty context 4
Sample phase 4
Empty context 5
Sample phase 5
70
55
35
c-Fos
70
55
35
Actin
b
Naive 1
Empty context 1
Sample phase 1
Naive 2
Empty context 2
Sample phase 2
Naive 3
Empty context 3
Sample phase 3
Naive 4
Empty context 4
Sample phase 4
Empty context 5
Sample phase 5
70
55
35
c-Fos
70
55
35
Actin
c
Naive 1
Empty context 1
Sample phase 1
Naive 2
Empty context 2
Sample phase 2
Naive 3
Empty context 3
Sample phase 3
Naive 4
Empty context 4
Sample phase 4
Empty context 5
Sample phase 5
55
35
25
c-Fos
55
35
Actin
d
Naive 1
Empty context 1
Sample phase 1
Naive 2
Empty context 2
Sample phase 2
Naive 3
Empty context 3
Sample phase 3
Naive 4
Empty context 4
Sample phase 4
Empty context 5
Sample phase 5
70
55
35
25
c-Fos
70
55
25
Actin
e
Naive 1
Empty context 1
Sample phase 1
Naive 2
Empty context 2
Sample phase 2
Naive 3
Empty context 3
Sample phase 3
Naive 4
Empty context 4
Sample phase 4
Empty context 5
Sample phase 5
70
55
35
25
c-Fos
70
55
25
Actin

**Figure S2: c-Fos levels increase in the anterior retrosplenial cortex 1 h after non-spatial Y-maze object recognition sample phase.** (A, B) Full-length immunoblots of the anterior retrosplenial cortex (aRSC) c-Fos (upper blots) and Actin (bottom blots). Boxes show the bands used for representative immunoblots. (C, D, E) Full-length immunoblots of the dorsal hippocampus (dHP) c-Fos (upper blots) and Actin (bottom blots). Boxes show the bands used for representative immunoblots.

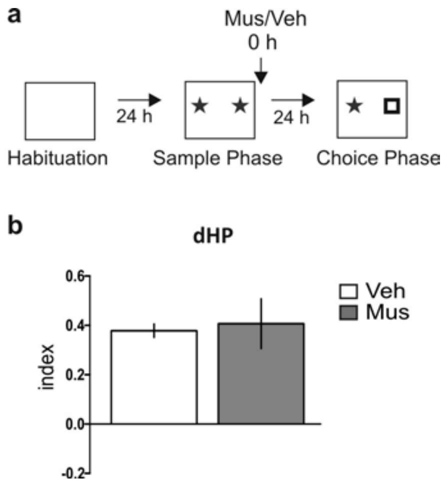


**Figure S3: The dorsal hippocampus is not required for the "what" memory formation even when the exploration time during sample phase is increased.** (A) Schematic representation of infusions and behavioral paradigm. (B) Memory index from animals infused with Mus (gray bar) or Veh (white bar) into the dHP immediately after an 8 min sample phase. Choice phase was performed 24 h later. Bars represent the memory index mean ± SEM. Mus vs. Veh; Two-tailed Student's t-test. n= 7.

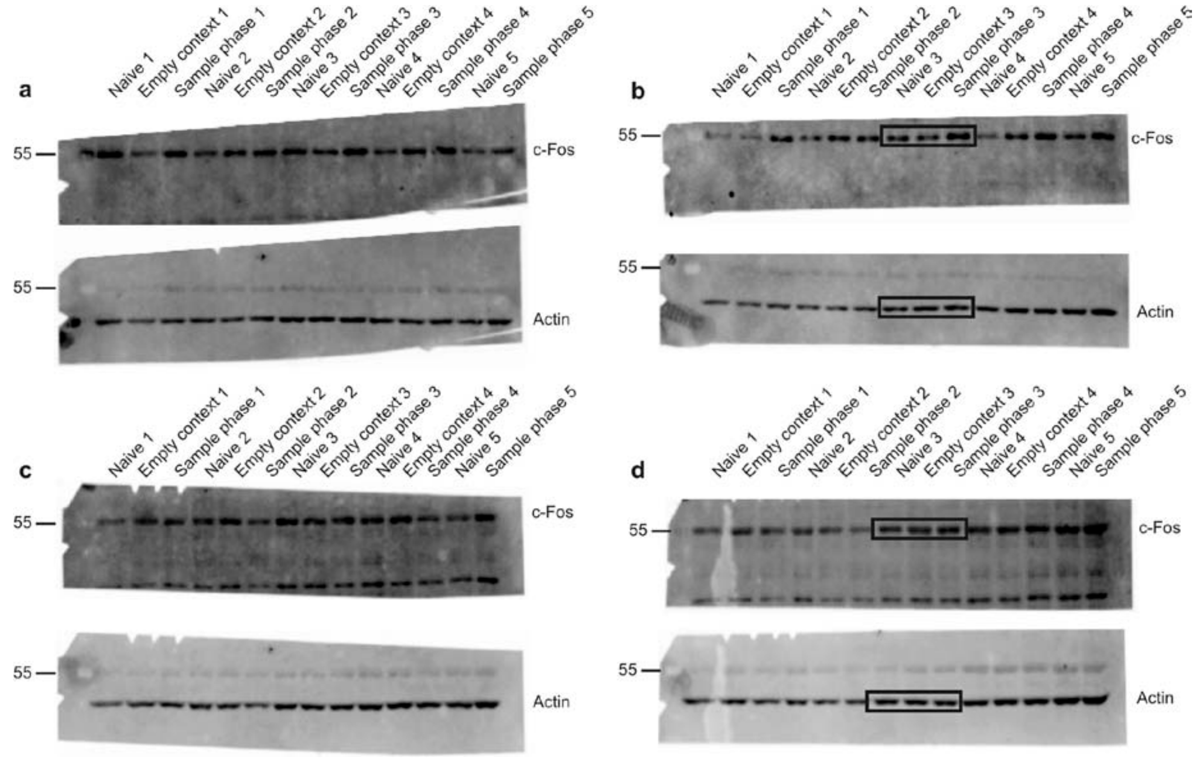


**Figure S4: c-Fos levels increase in the anterior retrosplenial cortex 1 h after object location sample phase.** (A, B) Full-length immunoblots of the aRSC c-Fos (upper blots) and Actin (bottom blots). Blots were cropped before developing western blot technique. Boxes show the bands used for representative immunoblots. (C, D) Full-length immunoblots of the dHP c-Fos (upper blots) and Actin (bottom blots). Blots were cropped before developing western blot technique. Boxes show the bands used for representative immunoblots.

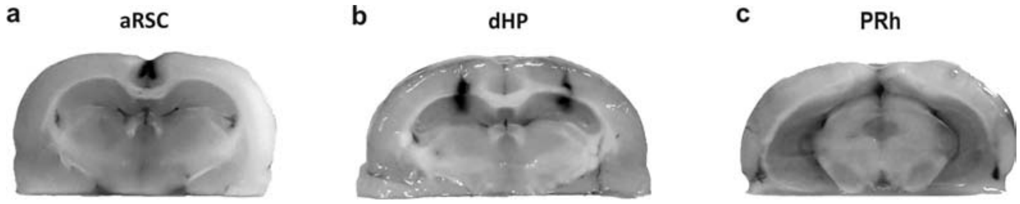


**Figure S5: Representative pictures showing the brain infusion area.** Animals were infused with 1 µl (A, B) or 0.5 µl (C) of 4% methylene blue (black) in saline into (A) aRSC, (B) dHP and (C) PRh

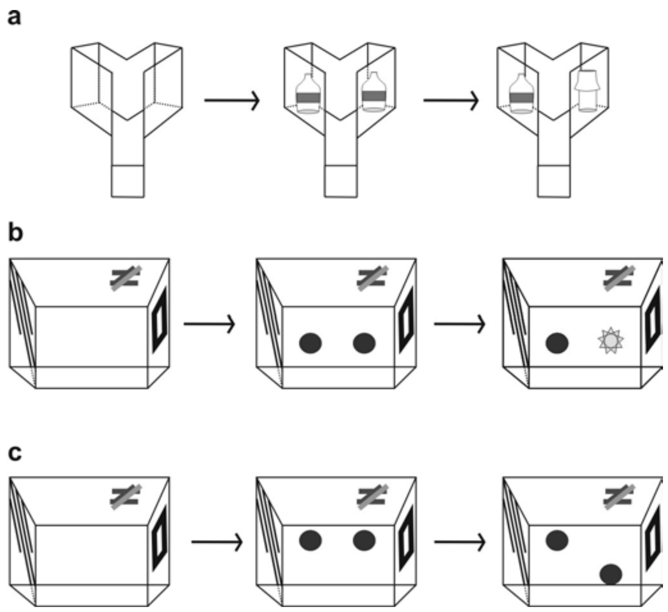


**Figure S6: Schematic illustration of the tasks used.** During habituation session animals were allowed to explore the empty maze. In the sample phase the rats explored two identical objects. During the choice phase animals were subject to a familiar object (sample session object) and a novel object (A, B) or object placement (C). (A) Front view of the Y-OR maze and objects position. (B) Top view of the SOR maze and objects position. (C) Top view of the OL maze and objects position.

| Figure | | n | total expl time SP | p-value SP | total expl time CP | p-value CP |
|---|---|---|---|---|---|---|
| **1b** | | | | 0.29 | | 0.44 |
| | Veh | 8 | 72.3 ± 11.3 | | 38.3 ± 7.4 | |
| | Mus | 8 | 58.3 ± 5.6 | | 30.8 ± 5.9 | |
| **1c** | | | | 0.40 | | 0.48 |
| | Veh | 5 | 72.6 ± 7.0 | | 31.6 ± 4.9 | |
| | Mus | 4 | 83.4 ± 9.8 | | 26.8 ± 4.0 | |
| **2a** | | | | 0.51 | | 0.81 |
| | Veh | 5 | 62.6 ± 13.0 | | 21.6 ± 4.6 | |
| | Mus | 8 | 71.9 ± 7.2 | | 23.0 ± 3.6 | |
| **2b** | | | | 0.68 | | 0.61 |
| | Veh | 6 | 62.7 ± 10.0 | | 25.8 ± 6.5 | |
| | Mus | 6 | 67.7 ± 5.9 | | 31.2 ± 7.9 | |
| **3b** | | | | 0.45 | | 0.94 |
| | Veh | 8 | 44.8 ± 4.8 | | 42.1 ± 4.8 | |
| | Mus | 6 | 51.5 ± 7.7 | | 42.7 ± 6.0 | |
| **3c** | | | | 0.08 | | 0.72 |
| | Veh | 7 | 58.7 ± 6.6 | | 30.4 ± 5.5 | |
| | Mus | 8 | 40.1 ± 7.0 | | 33.1 ± 4.7 | |
| **3e** | | | | 0.03 | | 0.32 |
| | Veh | 10 | 45.9 ± 5.7 | | 25.6 ± 2.9 | |
| | Mus | 11 | 60.6 ± 3.2 | | 29.6 ± 2.7 | |
| **3f** | | | | 0.06 | | 0.38 |
| | Veh | 8 | 71.9 ± 6.5 | | 38.3 ± 5.2 | |
| | Mus | 9 | 55.2 ± 5.0 | | 33.1 ± 2.8 | |
| **4a** | | | | 0.43 | | 0.80 |
| | Veh-Veh | 9 | 57.1 ± 5.9 | | 37.9 ± 4.9 | |
| | Veh-Mus | 5 | 53.6 ± 7.5 | | 38.8 ± 5.1 | |
| | Mus-Veh | 9 | 50.0 ± 4.1 | | 43.1 ± 6.1 | |
| | Mus-Mus | 9 | 62.2 ± 5.0 | | 42.9 ± 2.3 | |
| **4b** | | | | 0.68 | | 0.06 |
| | Veh-Veh | 11 | 64.9 ± 9.0 | | 48.6 ± 4.6 | |
| | Veh-Mus | 10 | 58.9 ± 7.9 | | 32.0 ± 5.1 | |
| | Mus-Veh | 9 | 56.7 ± 6.0 | | 35.2 ± 6.3 | |
| | Mus-Mus | 12 | 53.5 ± 4.3 | | 31.7 ± 4.5 | |
| **5b** | | | | 0.67 | | 0.16 |
| | Veh | 7 | 60.1 ± 4.4 | | 38.6 ± 6.6 | |
| | Mus | 6 | 65.2 ± 11.4 | | 24.7 ± 6.2 | |
| **5c** | | | | 0.68 | | 0.78 |
| | Veh | 7 | 58.3 ± 8.0 | | 32.9 ± 5.8 | |
| | Mus | 9 | 62.4 ± 6.3 | | 30.9 ± 4.0 | |
| **6b** | | | | 0.27 | | 0.18 |
| | Veh | 8 | 62.8 ± 9.6 | | 29.0 ± 3.6 | |
| | Mus | 8 | 84.1 ± 16 | | 37.8 ± 5.1 | |
| **6c** | | | | 1.00 | | 0.83 |
| | Veh | 12 | 113.6 ± 9.9 | | 40.3 ± 3.2 | |
| | Mus | 11 | 113.6 ± 8.5 | | 41.5 ± 4.0 | |
| **S1** | | | | 0.61 | | 0.56 |
| | Veh | 7 | 53.3 ± 5.9 | | 25.4 ± 6.0 | |
| | Mus | 5 | 57.3 ± 4.8 | | 20.8 ± 4.4 | |
| **S3** | | | | 0.13 | | 0.02 |
| | Veh | 7 | 111.3 ± 11.9 | | 52.1 ± 4.8 | |
| | Mus | 6 | 134.7 ± 8.8 | | 66.7 ± 2.9 | |

**Table 1: Total exploration time during sample and choice phases.** Data are expressed as mean ± SEM during sample (SP) and choice (CP) phases of each experiment for animals infused with vehicle (Veh) or muscimol (Mus).
